# Exosomal circular RNAs: Biogenesis, effect, and application in cardiovascular diseases

**DOI:** 10.3389/fcell.2022.948256

**Published:** 2022-08-09

**Authors:** Xiaoyi Hu, Hongran Qin, Yi Yan, Wenhui Wu, Sugang Gong, Lan Wang, Rong Jiang, Qinhua Zhao, Yuanyuan Sun, Qian Wang, Shang Wang, Hui Zhao, Jinming Liu, Ping Yuan

**Affiliations:** ^1^ Department of Cardio-Pulmonary Circulation, Shanghai Pulmonary Hospital, School of Medicine, Tongji University, Shanghai, China; ^2^ Department of Nuclear Radiation, Shanghai Pulmonary Hospital, School of Medicine, Tongji University, Shanghai, China; ^3^ Heart Center and Shanghai Institute of Pediatric Congenital Heart Disease, Shanghai Children’s Medical Center, National Children’s Medical Center, Shanghai Jiaotong University School of Medicine, Shanghai, China; ^4^ Institute of Bismuth Science, University of Shanghai for Science and Technology, Shanghai, China

**Keywords:** exosomes, circular RNAs, exosomal circRNAs, biogenesis and functions, cardiovascular diseases

## Abstract

As natural nanoparticles, exosomes regulate a wide range of biological processes *via* modulation of its components, including circular RNAs (circRNAs). CircRNAs are a novel class of closed-loop single-stranded RNAs with a wide distribution, and play diverse biological roles. Due to its stability in exosomes, exosomal circRNAs serve as biomarkers, pathogenic regulators and exert therapeutic potentials in some cardiovascular diseases, including atherosclerosis, acute coronary syndrome, ischemia/reperfusion injury, heart failure, and peripheral artery disease. In this review, we detailed the current knowledge on the biogenesis and functions of exosomes, circRNAs, and exosomal circRNAs, as well as their involvement in these cardiovascular diseases, providing novel insights into the diagnosis and treatment of these diseases.

## Introduction

Cardiovascular diseases (CVDs) are a group of heart and blood vessel diseases that are highly frequent among older adults, resulting in high mortality worldwide (GBD 2013 Mortality and Causes of Death Collaborators, 2015; Ma et al., 2020; Visseren et al., 2021). In recent years, the incidence of CVDs has shown a younger trend (Andersson and Vasan, 2018). However, the mechanisms behind CVDs pathogenesis remain largely unclear, making it difficult to improve the diagnostic and treatment strategies of these diseases (Li et al., 2018). Notably, exosomes have been studied extensively in the diagnosis, development, and treatment of CVDs (Ibrahim and Marban, 2016; Sahoo et al., 2021; Cui et al., 2022), including atrial fibrillation (AF), atherosclerosis (AS), diabetic cardiomyopathy, dilated cardiomyopathy, hypertension, heart failure (HF), ischemia/reperfusion (I/R) injury, myocardial infarction (MI), pulmonary hypertension (PH), and viral myocarditis. An elaborate review of exosomes might provide a novel insight into the diagnosis and treatment of these diseases.

Exosomes, 40–160 nm in diameter, are membrane-limited structures secreted by almost all living cells in both normal and pathological conditions (Kalluri and LeBleu, 2020). As crucial intercellular communication mediators, exosomes participate in the regulation of blood pressure, angiogenesis, cardiomyocyte hypertrophy, cardiac fibrosis, and apoptosis/survival (de Abreu et al., 2020). They are also potential biomarkers of CVDs due to their widespread prevalence in body fluids (Jansen et al., 2017). Furthermore, exosomes are expected to be a monotherapy in CVDs, considering that they are an essential component of the paracrine action of stem cell-based therapies (Lai et al., 2010).

Exosomes contain various components, including lipid (Flaherty et al., 2019), proteins (Ageta et al., 2018), DNA (Torralba et al., 2018), microRNA (miRNA), circular RNA (circRNA), and long noncoding RNA (lncRNA) (van Niel et al., 2018; Chen et al., 2019). In particular, circRNAs are nonlinear single-stranded RNAs (Memczak et al., 2013). The expression of circRNAs differs between healthy and pathological cardiovascular tissues, indicating that circRNAs are involved in the development of CVDs (Aufiero et al., 2019). Lines of evidence show that circRNAs participate in the pathogenesis of CVDs by binding miRNAs or RNA-binding proteins (RBPs), translating into proteins, and regulating transcription (Fan et al., 2017; Kishore et al., 2020; Rai et al., 2021). However, reviews of exosomal circRNAs and CVDs are currently quite limited.

In this review, we briefly summarized the current knowledge on the discovery history, biogenesis, and functions of exosomes and circRNA, as well as their participation in the pathogenesis of CVDs. Particularly, we detailed the significance and role of exosomal circRNAs in some CVDs, including AS, acute coronary syndrome (ACS), I/R injury, HF, and peripheral artery disease (PAD), emphasizing on their potential as promising diagnostic molecular markers and therapeutic targets.

## Overview of exosomes in CVDs

### Discovery and research history of exosomes

Exosomes were first discovered in sheep reticulocytes in 1983. The transferrin receptor in sheep reticulocytes were tracked during maturation *in vitro* with FITC- and 125I-labeled anti-transferrin-receptor antibodies; subsequently, the membrane protein transferrin receptor combined with vesicles, were later secreted into culture medium (Pan and Johnstone, 1983). In 1987, Johnstone et al. named those vesicles as exosomes. These vesicles contained various components, such as acetylcholinesterase, which was declined in sheep reticulocyte during maturation. Meanwhile, these vesicles exhibited characteristics of sheep red blood cells instead of white blood cells or platelets (Johnstone et al., 1987). However, exosomes were poorly explored during the next decade until 1996, when Raposo et al. discovered that exosomes produced from B lymphocytes participated in antigen presentation *in vivo* (Raposo et al., 1996). Since then, exosomes have received extensive attention.

In 2007, Valadi et al. suggested that both mRNAs and miRNAs were transferred between cells through exosomes and exerted functions in the recipient cells (Valadi et al., 2007). In 2010, exosomes were first studied as a potential agent for CVDs therapeutic intervention. Mesenchymal stem cell mediated its cardioprotective paracrine effect by secreting exosomes, subsequently reducing myocardial I/R injury (Lai et al., 2010). Afterwards, more and more researchers started to explore the relationship between exosomes and CVDs and a series of advances have been made in the research of exosomes in diagnosis, development, and treatment of CVDs (Ibrahim and Marban, 2016; Sahoo et al., 2021; Cui et al., 2022).

### Biogenesis of exosomes

**FIGURE 1:**
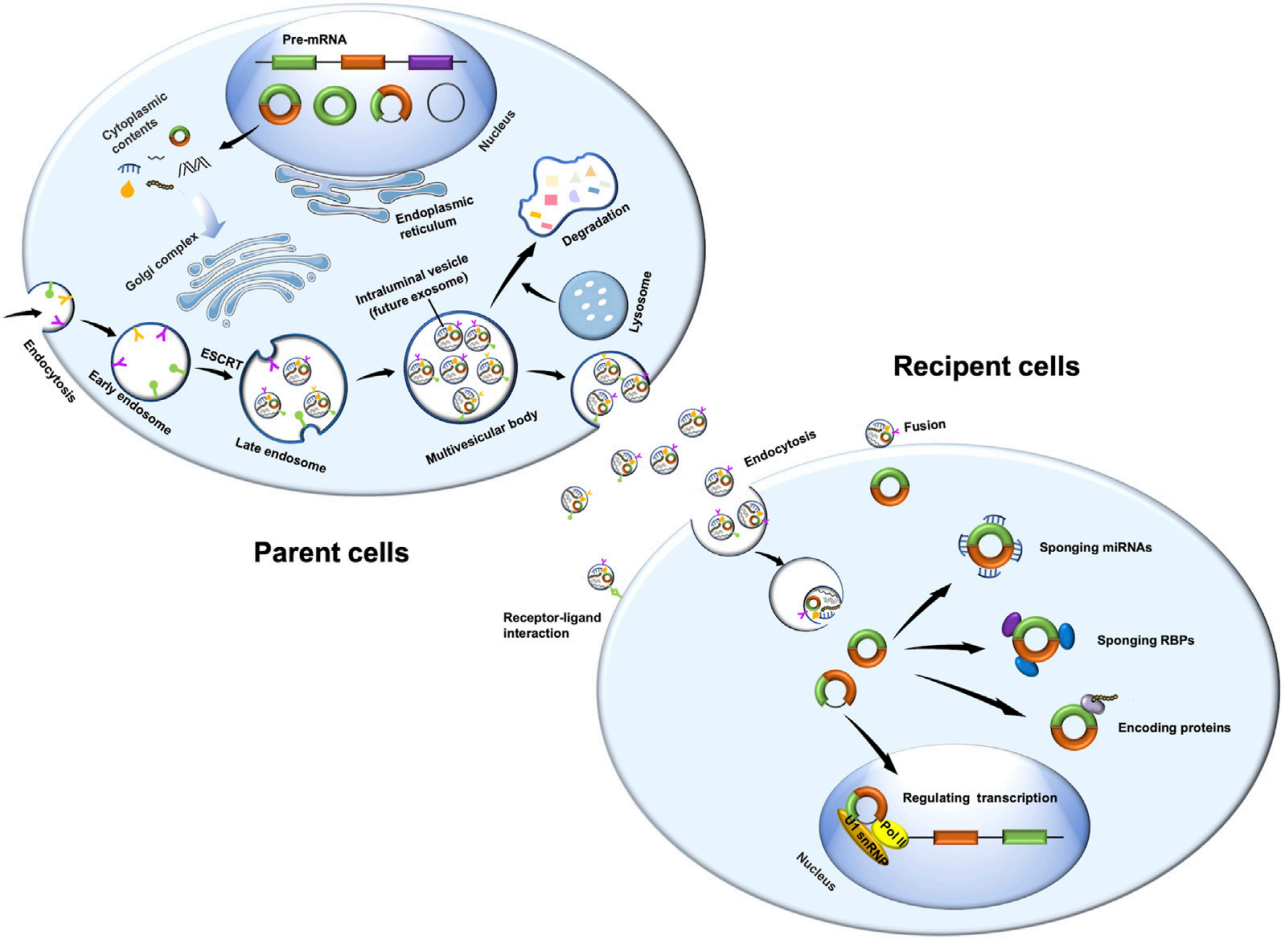
Biogenesis of exosomal circRNAs and its function in recipient cells. Exosomes are derived from endosomal system. Plasma membrane invagination results in the formation of early endosomes, whose membranes are then invaginated and sprouted to form intraluminal vesicles. At the same time, the early endosomes mature into multivesicular bodies. These intraluminal vesicles are essentially exosomes. Multivesicular bodies fuse with cell membrane, and exosomes are subsequently released to extracellular surroundings. CircRNAs are derived from pre-mRNA, which covalently connects both ends of a single RNA molecule during backsplicing to form a closed loop. CircRNAs have four main types, namely, intron-derived circRNAs, exonic circRNAs, exon-intron circRNAs, and intergenic circRNAs. MiRNAs and RBPs may regulate the sorting of circRNAs into exosomes. Exosomal circRNAs play important roles in recipient cells through sponging miRNAs or RBPs, encoding proteins, or regulating transcription. Pol II, RNA polymerase II; U1 snRNP, U1 small nuclear ribonucleoprotein.

Exosomes are derived from endosomal system (Figure 1). Plasma membrane invagination results in the formation of early endosomes, whose membranes are then invaginated and sprouted to form intraluminal vesicles. At the same time, the early endosomes mature into multivesicular bodies (MVBs), which contain numerous intraluminal vesicles in its internal cavity (Raposo and Stoorvogel, 2013; van Niel et al., 2018). Essentially, these intraluminal vesicles eventually become exosomes. MVBs fuse with cell membrane, and exosomes are subsequently released to extracellular surroundings. Exosome formation is controlled by two main mechanisms.

On one hand, the endosomal sorting complex required for transport (ESCRT) plays an essential role in exosome biogenesis. ESCRT contains five protein complexes: ESCRT-0, ESCRT-1, ESCRT-2, ESCRT3, and vacuolar protein sorting-associated protein 4. ESCRT-0, comprising hepatocyte growth factor-regulated tyrosine kinase substrate and signal transducing adaptor molecule, participates in the clustering of ubiquitinated cargoes. ESCRT-1 and ESCRT-2 lead to bud formation, ESCRT-3 drives vesicle scission, and vacuolar protein sorting-associated protein 4 is responsible for dissociation and recycling of the ESCRT machinery (Hurley and Hanson, 2010; Henne et al., 2013; Kowal et al., 2014). On the other hand, exosomes are formed in an ESCRT-independent manner. After depletion of key subunits of the four ESCRTs in HEp-2 cells, both early endosomes and MVBs remain differentiated, although the morphology of the endocytic pathway components may vary dramatically (Stuffers et al., 2009). CD63 (van Niel et al., 2011), CD81 (Perez-Hernandez et al., 2013), CD9 (Chairoungdua et al., 2010; Villarroya-Beltri et al., 2014), CD82 (Chairoungdua et al., 2010), and RAB31 (Wei et al., 2021) might be involved in ESCRT-independent endosomal sorting as well.

### Secretion of exosomes

The MVEs in cells have two different outcomes: MVBs could fuse with the lysosome to degrade their contents, or they could fuse with the plasma membrane and secrete exosomes into extracellular space (Kowal et al., 2014; van Niel et al., 2018). Some Rab GTPases are reported to regulate exosome secretion (Stenmark, 2009). For example, Rab27a, Rab27b, and Rab35 participate in the docking of MVEs at the plasma membrane (Ostrowski et al., 2010; Yang et al., 2019). Silencing of Rab27a or Rab27b could reduce exosome release without modifying the protein content or morphology of these exosomes. Slp4 and Slac2b are two effectors of Rab27, and silencing these effectors consequently reduces exosome secretion and phenocopies silencing of Rab27a and Rab27b (Ostrowski et al., 2010). Importantly, KIBRA is the upstream platform for exosome regulator, which controls exosome secretion by inhibiting the proteasomal degradation of Rab27a (Song et al., 2019). In addition, vesicle-membrane SNAREs (v-SNAREs) combined to target-membrane SNAREs (t-SNAREs), thereby regulating the fusion of MVEs to plasma membrane (Jahn and Scheller, 2006).

### Functions of exosomes

Exosome functions are mainly focused on three aspects. First, exosomes can transfer its contents, such as nucleic acids and proteins, to recipient cells to affect its function. Contents released from exosomes to biological fluids may also act as diagnostic biomarkers or predictors of disease progression and prognosis (Kalluri and LeBleu, 2020). Second, exosomes play significant roles in cell communications (Coumans et al., 2017). Finally, exosomes are an essential component of the paracrine action of stem cell–based therapies (Lai et al., 2010). Clinically, exosomes themselves or as vehicles for drug payload delivery are being actively explored as therapeutic agents (Andaloussi et al., 2013; de Abreu et al., 2020).

### Exosomes in CVDs

**FIGURE 2:**
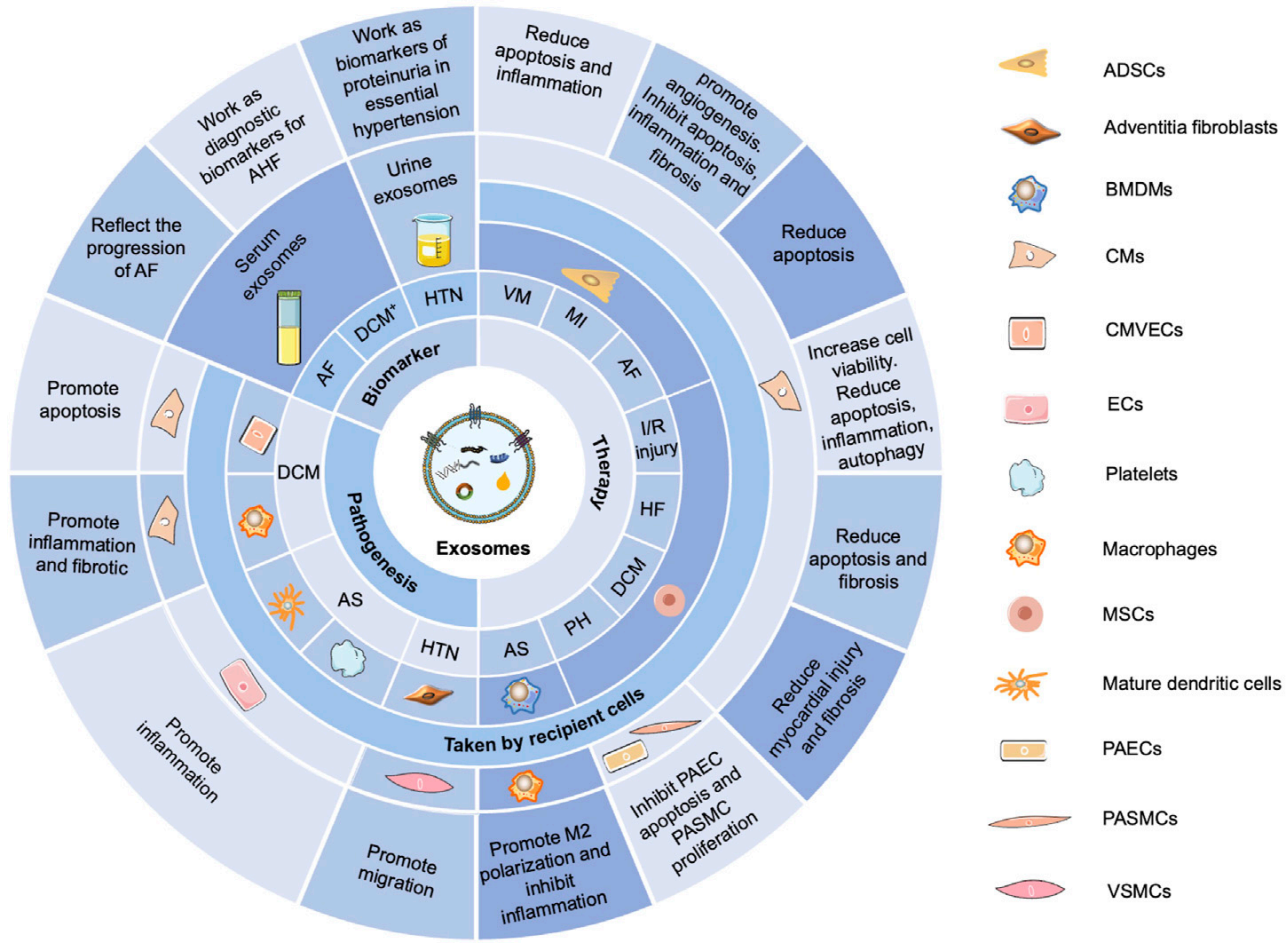
Functions of exosomes in CVDs. Exosomes in different body fluids, such as serum and urine, function as biomarkers to reflect the progression of many diseases, such as AF, dilated cardiomyopathy (DCM^+^), and hypertension (HTN). Exosomes can promote apoptosis, migration, inflammation, and cardiac fibrosis in diabetic cardiomyopathy (DCM), AS, and HTN. Exosomes derived from adipose-derived stem cells (ADSCs), mesenchymal stem cells (MSCs), or bone marrow–derived macrophages (BMDMs) exert therapeutic effects in viral myocarditis (VM), MI, AF, I/R injury, HF, DCM, PH, and AS. AHF, acute heart failure; CMVECs, cardiac microvascular endothelial cells; ECs, endothelial cells; PAECs, pulmonary arterial endothelial cells; PASMCs, pulmonary arterial smooth muscle cells; VSMCs, vascular smooth muscle cells.

The roles of exosomes in the diagnosis, development, and treatment of CVDs have already been studied extensively (Figure 2) (Ibrahim and Marban, 2016; Sahoo et al., 2021; Cui et al., 2022). Exosomes in different body fluids, such as serum and urine, may function as biomarkers to reflect the progression of many diseases, such as AF, dilated cardiomyopathy, and hypertension. As crucial intercellular communication mediators, exosomes could also promote apoptosis, migration, inflammation, and cardiac fibrosis in diabetic cardiomyopathy, AS, and hypertension. Notably, exosomes derived from adipose-derived stem cells, mesenchymal stem cells, or bone marrow-derived macrophages exert therapeutic effects in viral myocarditis, MI, AF, I/R injury, HF, diabetic cardiomyopathy, PH, and AS.

## Overview of circRNAs in CVDs

### Discovery and research history of circRNAs

Our previous review summarizes the discovery and research history of circRNAs (Wang et al., 2022). Briefly, these single-stranded closed RNAs were first identified in the genome of potato spindle in 1971 (Diener, 1971). In 2010, researchers discovered an extremely low expression of a circular isoform of the noncoding RNA ANRIL, which was associated with INK4/ARF expression and AS risk (Burd et al., 2010). With the advancement of RNA sequencing technology, circRNAs were finally detected in humans in 2012 (Salzman et al., 2012) and have been widely investigated since then.

### Biogenesis of circRNAs

As previously described (Wang et al., 2022), circRNAs are derived from pre-mRNA (Figure 1), which covalently connects both ends of a single RNA molecule during backsplicing to form a closed loop (Memczak et al., 2013). Owing to their unique loop structure, circRNAs are stable and resistant to RNase R (Jeck and Sharpless, 2014). CircRNAs are also endogenous, abundant, and conservative, with expression patterns specific to tissues, cell types, and developmental stages (Memczak et al., 2013; Geng et al., 2018; Kristensen et al., 2019).

CircRNAs have four main types, namely, intron-derived circRNAs, exonic circRNAs, exon-intron circRNAs, and intergenic circRNAs. In particular, intron-derived circRNAs contain circular intronic RNAs, excised group I introns, excised group II introns, excised tRNA introns, and intron lariats (Wang et al., 2016). Additionally, exonic circRNAs, which are produced primarily through an intron-pairing-driven circularization pattern, account for almost 80% of all circRNAs. Other circRNA biogenesis models include the exon-skipping model, RBP-dependent cyclization model, ciRNA formation model, and variable cyclization model (Li B. et al., 2021).

### Functions of circRNAs

CircRNAs participate in physiological processes *via* different molecular pathways (Figure 1). First, circRNAs may function as a sponge for miRNAs or competitive endogenous RNAs, thereby inhibiting miRNA expression and thus enhancing miRNA-targeted mRNAs (Hansen et al., 2013). Second, circRNAs may interact with RBPs and regulate mRNA expression by altering the splicing patterns or mRNA stability (Abdelmohsen et al., 2017; Du et al., 2017; Yang et al., 2017). Third, circRNAs may influence transcription by interacting with the RNA polymerase II machinery and U1 small nuclear ribonucleoprotein in the nucleus (Li Y. et al., 2015; Ng et al., 2016). In addition, circRNAs may regulate the transcription of their parent genes by competing with linear mRNA splicing (Li Z. et al., 2015; Xu et al., 2020). Finally, circRNAs could be translated into proteins with ribosomes (Legnini et al., 2017; Pamudurti et al., 2017; Begum et al., 2018; Yang et al., 2018).

### CircRNAs in CVDs

**FIGURE 3:**
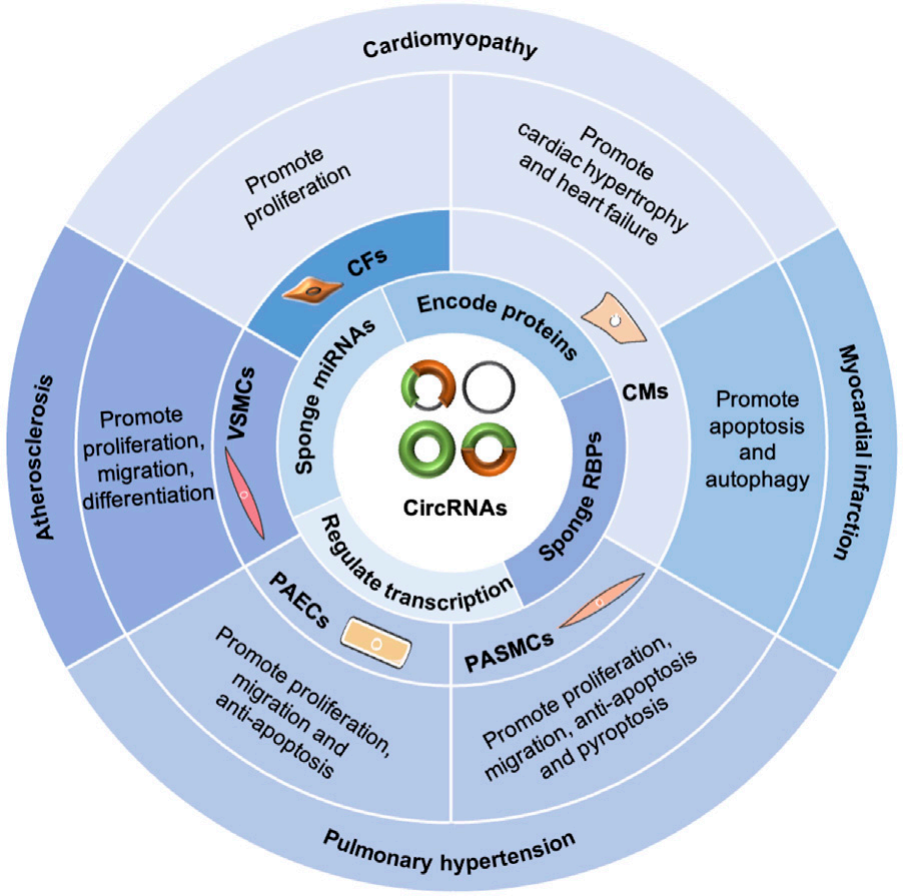
Functions of circRNAs in CVDs. CircRNAs exert functions in CVDs by sponging miRNAs and RBPs, encoding proteins, and regulating transcription. In the cardiovascular system, circRNAs are involved with cardiac fibroblasts (CFs), cardiomyocytes (CMs), pulmonary arterial smooth muscle cells (PASMCs), pulmonary arterial endothelial cells (PAECs), and vascular smooth muscle cells (VSMCs), playing important roles in the pathogenesis of cardiomyopathy, myocardial infarction, pulmonary hypertension, and atherosclerosis.

Generally, circRNAs exert functions in CVDs (Figure 3) by sponging miRNAs and RBPs, encoding proteins, and regulating transcription (Fan et al., 2017; Kishore et al., 2020; Rai et al., 2021). In the cardiovascular system, circRNAs are associated with cardiac fibroblasts (CFs), cardiomyocytes (CMs), pulmonary arterial smooth muscle cells, pulmonary arterial endothelial cells, and vascular smooth muscle cells (VSMCs), playing important roles in the pathogenesis of cardiomyopathy, MI, PH, and AS (Kishore et al., 2020). Furthermore, circRNAs can serve as potential diagnostic and prognostic biomarkers for CVDs (Li B. et al., 2020; Yuan et al., 2021).

## Overview of exosomal circular RNAs in CVDs

### Selection mechanism of circRNAs into exosomes

Two pathways may influence the selection of circRNAs into exosomes. First, miRNAs might be responsible for the process. It was demonstrated that miR-7 mimics treatment for the cells led to a significant reduction of its competitive endogenous sponge circCDR1as in exosomes (Hansen et al., 2013; Li Y. et al., 2015), suggesting that sorting of circRNAs to exosomes was regulated, at least in part, by changes of associated miRNA levels in producer cells. In addition, miRNAs could mediate the intracellular degradation of circRNAs, thus causing the decrease of the expression of circRNAs in exosomes. For example, miR-671-AGO2 was reported to mediate the degradation of circCDR1as (Hansen et al., 2011) in source cells, which might be responsible for the reduction of circCDR1as in exosomes. Second, RBPs may also regulate the selection of circRNAs into exosomes by binding to specific sequences (Schickel et al., 2008; Frank et al., 2010). According to the study of Villarroya-Beltri et al., RBP hnRNPA2B1 binds exosomal miRNAs through the recognition of specific sequence motifs and controls their loading into exosomes (Villarroya-Beltri et al., 2013). There might be similar mechanisms modulating the selection of circRNA into exosomes as that of miRNAs. Existing evidence indicated that RBPs were abundant in exosomes from DKs-8 cell and could bind circFAT1 for its loading into exosomes (Dou et al., 2016). However, the specific selection mechanism of circRNAs into exosomes remains unclear and warrants further investigation.

### Exosomal circRNAs in CVDs

**FIGURE 4:**
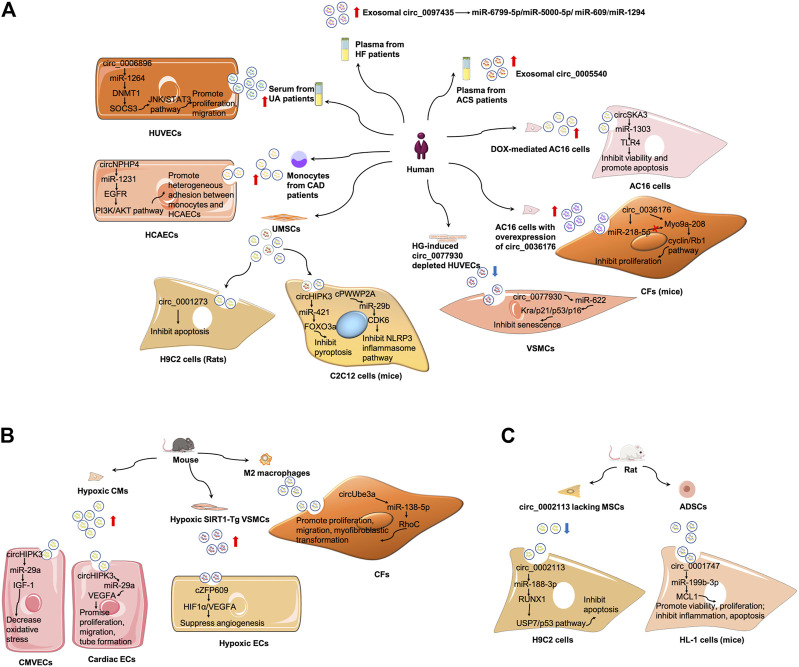
Mechanism of exosomal circRNAs in CVDs. **(A)** Summary of studies of exosomal circRNAs in human CVDs. **(B)** Summary of studies of exosomal circRNAs in mouse CVDs. **(C)** Summary of studies of exosomal circRNAs in rat CVDs. ADSCs, adipose-derived stem cells; CAD, coronary artery disease; CMVECs, cardiac microvascular endothelial cells; ECs, endothelial cells; HCAECs, human coronary artery endothelial cells; HG, hyperglycemia; HUVECs, human umbilical vein endothelial cells; MSCs, mesenchymal stem cells; UA, unstable/vulnerable plaque atherosclerosis; VSMCs, vascular smooth muscle cells.

Exosomal circRNAs were first studied in CVDs in 2019 (Ge et al., 2019). Ge et al. discovered alterations of circRNA expression in mouse cardiac exosomes after I/R injury and identified some potential targets and pathways involved in I/R injury (Ge et al., 2019). Since then, the crucial roles of exosomal circRNAs in CVDs have been widely explored. In this section, the roles of exosomal circRNAs as biomarkers, pathogenetic mediators and therapeutic potentials based on current knowledge will be revealed in the context of CVDs (Figure 4; Tables 1, 2).

**TABLE 1:** The biomarkers of serum/plasma exosomal circRNAs in cardiovascular diseases.

CircRNA	Expression	Disease	Function	References
circ_0006896	↑	AS	Diagnostic	Wen et al. (2021)
circ_0005540	↑	CAD	Diagnostic	Wu et al. (2020)
circNPHP4	↑	CAD	Diagnostic	Xiong et al. (2021)

AS, atherosclerosis; CAD, coronary artery disease; CircRNA, circular RNA.

**TABLE 2:** The therapeutic role of stem cell-derived exosomal circRNAs in cardiovascular diseases.

CircRNA	Stem cells	Recipient cells	Disease	Function	References
circ_0001273	UMSCs	CMs	MI	Inhibit apoptosis	Li B. et al. (2020)
circ_0002113	MSCs	H9C2 cells	I/R injury	Inhibit apoptosis	Tian et al. (2021)
circ_0001747	ADSCs	HL-1 cells	I/R injury	Promote viability and proliferation; inhibit inflammation and apoptosis	Zhou et al. (2022)
cPWWP2A	UMSCs	C2C12 cells	PAD	Inhibit NLRP3 inflammasome pathway	Wang et al. (2021a)
circHIPK3	UMSCs	C2C12 cells	PAD	Inhibit pyroptosis	Yan et al. (2020)

ADSCs, adipose-derived stem cells; CircRNA, circular RNA; I/R, ischemia/reperfusion; MI, myocardial infarction; MSCs, mesenchymal stem cells; PAD, peripheral artery disease; UMSCs, umbilical cord mesenchymal stem cells.

#### Exosomal circRNAs in AS

AS is a chronic inflammatory disease of the arterial wall, characterized by the formation of plaques containing lipid, connective tissue, and immune cells in the intima of large and medium arteries (Engelen et al., 2022). AS is also the common cause of MI and stroke. Endothelial cell (EC) and VSMC dysfunction and inflammatory cell infiltration are all major contributors in AS development (Falk, 2006).

(1) Exosomal circRNAs serve as biomarkers of AS (Vilades et al., 2020; Wen et al., 2021). Serum exosomes from patients with unstable/vulnerable atherosclerotic plaque showed a markedly upregulated expression of circ_0006896 compared with those from patients with stable atherosclerotic plaque. The serum exosomal circ_006896 level was also positively related to the levels of triglyceride, low-density lipoprotein cholesterol, and C-reactive protein but negatively associated with albumin level, indicative of an association between circ_0006896 with plaque instability (Wen et al., 2021).
(2) Exosomal circRNAs are involved in AS pathogenesis. Compared with the serum levels of circ_0006896 in the exosomes from patients with stable plaque, the higher expression in patients with vulnerable plaque could increase DNMT1 expression in human umbilical vein endothelial cells by directly binding to miR-1264 (Wen et al., 2021). DNMT1 was reported to regulate DNA methylation and could methylate the promoter region of SOCS3 and repress its gene expression (Boosani et al., 2015). The reduced expression of SOCS3 then led to the increase of STAT3 phosphorylation, and activated JNK/STAT3 pathway. Therefore, circ_0006896 facilitated the proliferation and migration of human umbilical vein endothelial cells *via* the miR-1264/DNMT1/SOCS3 pathway in JNK/STAT3-dependent manner (Wen et al., 2021). Wang et al. found that the expression of circ_0077930 from hyperglycemia-induced human umbilical vein endothelial cells was upregulated in exosomes, which was abolished by circ_0077930 knockdown. Treating VSMCs with circ_0077930-depleted exosomes could increase the expression of miR-622 and reduce the expression of Kras, as well as aging-related proteins p21, p53, and p16. Meanwhile, the lactate dehydrogenase activity decreased, but the superoxide dismutase activity and anti-oxidative stress marker increased. Therefore, circ_0077930-depleted exosomes fail to induce VSMC senescence (Wang Y. et al., 2020).

#### Exosomal circRNAs in ACS

ACS is a group of clinical syndromes including ST segment elevation MI, acute non-ST segment elevation MI, and unstable angina pectoris. The pathophysiological underpinning of ACS is the rupture or invasion of coronary atherosclerotic plaques, followed by complete or incomplete occlusive thrombosis. Prompt and appropriate treatment for ACS patients is crucial to reduce mortality and improve prognosis (Libby, 2013).

(1) Exosomal circRNAs serve as biomarkers in ACS. Wu et al. screened differentially expressed exosomal circRNAs in the plasma of patients with coronary artery disease (CAD) and found 164 upregulated and 191 downregulated circRNAs. Moreover, exosomal circ_0005540 was increased in patients with CAD (105 patients with CAD vs 86 non-CAD controls). However, further studies are still needed to confirm whether plasma-derived exosomal circ_0005540 could be used as a diagnostic biomarker for CAD (Wu et al., 2020). In another study, circNPHP4 expression was considerably evaluated in exosomes isolated from the monocytes of patients with CAD; results suggested that serum circNPHP4 upregulation predicted aggressive clinicopathological characteristics in patients with CAD (Xiong et al., 2021).
(2) Exosomal circRNAs participate in ACS pathogenesis. In particular, circNPHP4 expression positively correlated with the expression of its parent gene NPHP4 in monocytes from both patients with CAD and control subjects. Functional assays indicated that exosomal circNPHP4 knockdown inhibited heterogeneous adhesion between monocytes and human coronary artery endothelial cells, alongside with the reduction of adhesive molecules (ICAM-1 and VCAM-1). Mechanically, exosomal circNPHP4 may promote the EGFR/PI3K/AKT pathway by inhibiting miR-1231 in human coronary artery endothelial cells to affect its recruitment of monocytes in patients with CAD (Xiong et al., 2021). Wang et al. found that circHIPK3 was upregulated in exosomes derived from CMs under hypoxic conditions (Wang et al., 2019a; Wang S. et al., 2020). CircHIPK3 could be internalized by cardiac microvascular endothelial cells *via* exosomes. Exosomal circHIPK3 from hypoxic CMs could increase IGF-1 expression by sponging miR-29a, thereby ameliorating oxidative stress-induced cardiac microvascular endothelial cell dysfunction (Wang et al., 2019a). In cardiac ECs treated with exosomes from hypoxia induced CMs, the level of circHIPK3 increased. Furthermore, CMs-derived exosomes under hypoxic conditions promoted cardiac EC proliferation, migration, and tube formation *via* the circHIPK3/miR-29a/VEGFA axis; they also promoted neovascularization after MI and ameliorated myocardial fibrosis *in vivo* (Wang Y. et al., 2020). Moreover, M2 macrophage-derived exosomes transferred circUbe3a to CFs to sponge miR-138-5p and inhibit its expression, and then increase RhoC expression, thereby promoting cell proliferation, migration, and myofibroblastic transformation. Therefore, exosomal circUbe3a derived from M2 macrophages might exacerbate myocardial fibrosis after acute MI (Wang et al., 2021a).
(3) Exosomal circRNAs have therapeutic potentials in ACS. Circ_0001273 in exosomes was higher than that in its parent cells, that is, umbilical cord mesenchymal stem cell (UMSCs), at each time point. It was reported that cardiac circ_0001273 was downregulated in MI rat model. UMSC-derived exosomes promoted MI repair by transmitting circ_0001273, as evidenced by the abrogation of the beneficial effect after si-circ-0001273 UMSC-derived exosome treatment. In addition, circ_0001273 from UMSC-derived exosomes repressed CM apoptosis, thereby promoting MI repair (Li C. X. et al., 2020).

#### Exosomal circRNAs in I/R injury

Myocardial I/R injury refers to the injury of myocardial tissues after partial or complete acute occlusion of the coronary artery (Kalogeris et al., 2016). Myocardial I/R injury occurs frequently after thrombolysis or percutaneous coronary intervention in clinical practice, probably resulting from an oxygen-derived free-radical burst, quick physiological pH recovery, insulin resistance, intracellular calcium overload, mitochondrial damage, or inflammatory insults (Wu et al., 2018; Soares et al., 2019).

(1) Although there are no studies on the role of exosomal circRNAs as biomarkers in I/R injury, Ge et al. discovered 185 differentially expressed exosomal circRNAs, with 66 upregulated and 119 downregulated, in exosomes derived from a murine heart after I/R injury. Gene ontology and pathway analyses indicated that upregulated circRNAs were possibly related to inflammatory regulation after cardiac I/R injury, whereas downregulated circRNAs might participate in regulating fibrotic response and cardiac dysfunction (Ge et al., 2019).
(2) Exosomal circRNAs have therapeutic potentials in I/R injury. Exosomes derived from circ_0002113 lacking mesenchymal stem cells reduced myocardial injury by sponging miR-188-3p to regulate RUNX1 nuclear translocation. The circ_0002113/miR-188-3p/RUNX1 axis regulated H/R-induced H9C2 cell apoptosis in a USP7/p53 dependent manner, serving as a novel strategy for myocardial I/R injury treatment (Tian et al., 2021). In another study, circ_0001747 was downregulated in murine H/R injury myocardial cells (HL-1 cells). H/R exposure restrained cell viability and proliferation, and induced cell inflammation and apoptosis. However, exosomes with have high amounts of circ_0001747 from adipose-derived stem cells largely attenuate H/R-induced dysfunction in HL-1 cells by targeting the miR-199b-3p/MCL1 axis (Zhou et al., 2022).

#### Exosomal circRNAs in HF

HF is a dyscirculatory syndrome caused by cardiac systolic and/or diastolic dysfunction, resulting in insufficient perfusion of venous blood pool and arterial blood. This disorder manifests as pulmonary congestion and vena cava thrombosis. HF is not an independent disease but a terminal stage in CVD development. Most cases of HF begin with the left side, which first presents as congestion of the pulmonary circulation (Tamargo and Lopez-sendon, 2011).

(1) So far, there have been no studies on the role of exosomal circRNAs as biomarkers in HF. However, Han et al. screened differentially expressed circRNAs in peripheral blood samples from five patients with HF and found 56 differentially expressed circRNAs, of which 29 were upregulated and 27 were downregulated relative to that of control subjects. They further examined circ_0097435 expression in the plasma exosomes of 15 patients with HF and 15 healthy volunteers. It turned out that circ_0097435 levels were markedly higher in exosomes from patients with HF (Han et al., 2020). However, the potential of circ_0097435 as a biomarker in HF remains unconfirmed.
(2) Exosomal circRNAs are involved in HF pathogenesis. The overexpression of circ_0097435 promoted apoptosis in CMs, which was abrogated by circ_0097435 knockdown (Han et al., 2020). Further RNA-pulldown and AGO2-immunoprecipitation experiments demonstrated that circ_0097435 might serve as a sponge for multiple miRNAs, such as miR-6799-5p, miR-5000-5p, miR-609, and miR-1294. Doxorubicin (DOX) is a widely used anticancer drug. It was shown that circSKA3 was overexpressed in exosomes from DOX-mediated AC16 cells, which could be further internalized by surrounding untreated AC16 cells, contributing to DOX-induced cardiotoxicity through the miR-1303/TLR4 axis. The knockdown of circSKA3 could partially reverses the increase of cell apoptosis and decrease of cell viability in AC16 cells in response to DOX administration (Li L. et al., 2021). In another study, circ_0036176 was upregulated in the myocardium of patients with HF, and it was abundant in exosomes derived from human AC16 cardiomyocytes with overexpression of circ_0036176 (OE-circ_0036176-exo). OE-circ_0036176-exo could inhibit the proliferation by translating Myo9a-208 protein to suppress the expression of CCND1, CCNE1, CDK9, and p-RB1 in CFs. While miR-218-5p could bind to circ_0036176 to suppress Myo9a-208 expression at the transcriptional level, thereby attenuating the inhibitory effect of circ_0036176 on mouse CF proliferation (Guo et al., 2022).

#### Exosomal circRNAs in PAD

PAD is a syndrome of peripheral circulatory dysfunction characterized by the narrowing, occlusion, or tumor-like dilation of main arteries and their branch vessels other than the cardiac and cerebral arteries. One of the leading causes of PAD is AS, which may lead to acute lower-limb ischemia (Morley et al., 2018). The disease has an insidious beginning and could be asymptomatic in the early stages. Furthermore, PAD can possibly result in intermittent claudication, ischemic rest discomfort, ulcers, and prolonged treatment, and gangrene or even amputation in extreme circumstances with an unfavorable prognosis (Aboyans et al., 2018).

(1) Exosomal circRNAs act as mediators in PAD pathogenesis. Dou et al. revealed that cZFP609 was highly expressed in human silent information regulator 1 (SIRT1)-overexpressing VSMCs, as well as in its exosome under hypoxic conditions (Dou et al., 2020). SIRT1 is a NAD^+^-dependent histone deacetylase that could mediate endothelial angiogenic functions during vascular growth (Potente et al., 2007). It was demonstrated that exosomes from SIRT1-overexpressing VSMCs could transfer cZFP609 to ECs. The cZFP609 detained the HIF1α in the cytoplasm *via* its interaction with HIF1α, thereby inhibiting VEGFA expression and suppressing endothelial angiogenesis under hypoxic conditions.
(2) Exosomal circRNAs have therapeutic potentials in PAD. By transferring cPWWP2A, UMSC-Exos could increase the blood flow of ischemic hindlimb and promote skeletal muscle repair after injury*.* In addition, cPWWP2A inhibited the tumor suppressor Rb1-mediated NLRP3 inflammasome pathway through the miR-29b/CDK6 axis in C2C12 cells (Wang et al., 2021b). In another research, UMSC-Exos could promote ischemic hindlimb repair and prevent skeletal muscle pyroptosis by delivering circHIPK3 *in vivo.* UMSC-Exos also prevented lipopolysaccharide-induced C2C12 cells from pyroptosis *via* the circHIPK3/miR-421/FOXO3a axis (Yan et al., 2020).

#### Exosomal circRNAs in other CVDs

Exosomal circRNAs might also be involved in other types of CVDs (eg. hypertension, PH and AF) based on the evidences on the investigation of exosomes and circRNAs in the disease settings, despite the lack of researches on the direct role of exosomal circRNAs in these diseases (Ibrahim and Marban, 2016; Fan et al., 2017; Kishore et al., 2020; Rai et al., 2021; Sahoo et al., 2021; Cui et al., 2022). For example, the therapeutic effects of exosomes in PH have been extensively studied (Willis et al., 2018; Oh et al., 2022). Furthermore, Xiang et al. have reported the potential of exosomes as therapeutic target and clinical biomarker in AF (Xiang et al., 2022). Several researchers also reviewed the roles of exosomes in hypertension (Arishe et al., 2021), as well as the roles of circRNAs in hypertension and PH (Sekar, 2019; Zaiou, 2019; Ali et al., 2022; Wang et al., 2022). Moreover, the expression profiles of circRNAs in AF patients have been identified (Zhu et al., 2020). Therefore, the specific roles of exosomal circRNAs warrant further investigation in hypertension, PH and AF as well.

## Conclusion

Due to their unique features and high specificity, exosomal circRNAs are stable, and present in diverse extracellular fluids, such as blood and urine, making it an ideal diagnostic biomarker and therapeutic targets in diseases (Guo et al., 2021). However, the studies of circRNAs in exosomes are still incomplete compared to other non-coding RNAs, such as miRNAs and lncRNAs. Furthermore, many challenges and difficulties exist in the clinical applications of exosomal circRNAs. First, it is difficult to detect circRNAs in exosomes with accurate methods due to their low abundance in some cases (Boriachek et al., 2018). Second, since circRNAs and their liner mRNA have some overlapped sequences, the expression and function of circRNAs cannot be precisely evaluated. Third, there is no standardized method for isolating and processing exosomes (Wang et al., 2019b). Therefore, advanced technologies and methods are needed to elucidate the molecular mechanism of exosomal circRNAs.

Exosomal circRNAs are emerging as a new area of research in CVDs. Exosomal circRNAs exert multiple functions related to cell viability, proliferation, apoptosis, migration, invasion, tube formation, inflammation, and adhesion. They also serve as biomarkers for the diagnosis and progression of CVDs. In addition, some exosomal circRNAs from stem cells have therapeutic potentials in CVDs. Future studies can focus on the following directions: *1*) to explore the roles of exosomal circRNAs in other types of CVDs, such as hypertension, PH, and AF; *2*) to reveal the specific mechanism of exosomal circRNAs in the pathogenesis of CVDs; *3*) to evaluate the efficacy of different exosomal circRNAs derived from various stem cells in CVDs. A full understanding of the relationship between exosomal circRNAs and CVDs is important to generate novel ideas for the diagnosis and treatment of CVDs.
